# From balance to perception: vestibular-cognitive integration predicts signal discrimination in a Multirule Visual Monitoring Task

**DOI:** 10.3389/fpsyg.2026.1759775

**Published:** 2026-03-23

**Authors:** Yuan Zhou, Yuxuan Chen, Jing Zhang, Ying Long, Jianbo Lei, Ming Chang

**Affiliations:** 1 School of Psychology, Shaanxi Normal University, Xi'an, China; 2 Shaanxi Provincial Key Laboratory of Behavior and Cognitive Neuroscience, Xi'an, China; 3 College of Sociology, Nankai University, Tianjin, China; 4 School of Physical Education, Shaanxi Normal University, Xi'an, China

**Keywords:** attentional breadth, interoception, multisensory integration, perceptual sensitivity, vestibular processing, vigilance

## Abstract

**Objective:**

The vestibular system has been hypothesized to serve as a cognitive anchor for spatial processing, yet its functional contribution under conditions of high cognitive load remains unclear. This study investigated how vestibular function interacts with attentional processes, specifically attentional breadth, to support perceptual discrimination during a demanding Multirule Visual Monitoring Task.

**Methods:**

Participants performed a visual discrimination task with varying time pressure and load. Vestibular function, attentional shifting, and attentional breadth were assessed. Binary logistic regression and ROC analyses evaluated these factors’ ability to distinguish between high- and low-performance groups.

**Results:**

In-sample classification analyses indicated that models incorporating both vestibular function and attentional breadth achieved higher classification performance in both simple (AUC = 0.961) and difficult (AUC = 0.945) task conditions than single-predictor model. Conversely, analysis of variance based on continuous performance scores revealed no significant interaction between vestibular function and attention. This divergence suggests that the joint contribution may be nonlinear or threshold-dependent, primarily captured through categorical classification rather than linear modulation of performance.

**Conclusion:**

The findings are consistent with theoretical accounts of vestibular-cognitive interactions and indicate a joint contribution of vestibular function and attentional breadth to perceptual performance. The divergence between categorical and continuous analyses suggests this contribution may be nonlinear or threshold-dependent. Future work employing cross-validation and direct tests of nonlinear models is needed to further elucidate this relationship.

## 1 Introduction

Human perception is an active, constructive process whereby the brain synthesizes information from multiple sensory channels to form stable and coherent representations of the environment (Stein et al., 2014; Chen and Spence, 2017). Multisensory integration, the process by which the brain combines information from distinct sensory modalities, optimizes adaptive behavior by enhancing perceptual precision, accelerating responses, and increasing decision robustness (Ernst and Banks, 2002; Durk et al., 2010; Olaf et al., 2015). It improves signal detection thresholds and optimizes behavioral outputs through statistically optimal cue weighting (Fetsch et al., 2013; Fetsch and Noppeney, 2023). While research has historically prioritized exteroceptive sensory streams (e.g., audiovisual interactions), internal physiological feedback (interoceptive signals) is now recognized as critically shaping cognition (Zeng et al., 2023), perceptual awareness, and self-representation (Erik et al., 2016; Kirsch and Kunde, 2022). Interoceptive signals dynamically modulate cortical processes, such as inferring causality in sensory events (Keisuke et al., 2013) and anchoring percepts to embodied priors (Anil, 2013; Barrett and Simmons, 2015).

Building upon the role of interoception, the vestibular system serves as a paradigmatic model for understanding how bodily signals scaffold cognitive processes. The traditional view of the vestibular system as a simple sensor for balance and spatial orientation has been revised. Neuroanatomical studies reveal extensive ascending pathways from vestibular nuclei to a widespread cortical network, including the hippocampus, parahippocampal gyrus, temporo-parietal junction, and insula—regions involved in memory, spatial navigation, and bodily self-consciousness (Eulenburg et al., 2011; Hitier et al., 2014). This anatomical convergence suggests vestibular information is deeply embedded within neural substrates of cognition.

We propose that the vestibular system functions as a fundamental spatial cognitive anchor. By providing a continuous, gravity-referenced signal of self-motion and orientation, it generates a stable egocentric reference frame upon which other sensory and cognitive information can be mapped (Pfeiffer et al., 2014). This hypothesis extends the cognitive-vestibular compensation hypothesis (Lacroix et al., 2021), which posits that efficient vestibular processing frees cognitive resources for other tasks. While the compensation hypothesis focuses on the consequence of vestibular efficiency (resource liberation), our “spatial cognitive anchor” hypothesis addresses the underlying mechanism: the continuous, automated provision of a stable spatial reference that reduces the internal computational load required for spatial updating, thereby optimizing resource allocation. Clinical evidence strongly supports this integrative view. Patients with bilateral vestibular loss exhibit deficits in spatial navigation, memory, and mental rotation (Popp et al., 2017; Schöberl et al., 2021) and show hippocampal atrophy (Brandt et al., 2005). Furthermore, Galvanic Vestibular Stimulation in healthy participants modulates performance on tasks requiring egocentric spatial transformations, demonstrating a causal link between vestibular signals and spatial cognition (Ferrè et al., 2012; Diane et al., 2015).

The mechanism linking vestibular function to cognition can be understood through cognitive resource allocation. A stable vestibular signal may automate spatial grounding, reducing the cognitive load required for continuously updating one’s position in space (Smith, 2017). This liberated attentional capacity can then be allocated to external task demands. Conversely, vestibular dysfunction may force the brain to dedicate excessive resources to compensate for an unstable internal reference, increasing cognitive load and impairing performance on concurrent tasks—a phenomenon observed in dual-task paradigms (Furman et al., 2012; Diane et al., 2015). Therefore, vestibular function is conceptualized as a process of continuous spatial calibration that, by automating stable self-representation, optimizes the allocation of limited cognitive resources for higher-order processing.

Despite this compelling theoretical framework, direct empirical evidence within the context of complex, rule-based tasks imposing high cognitive load remains scarce. Most existing studies have examined balance and cognitive functions in isolation, failing to capture their dynamic interplay (Lacroix et al., 2021; Smith, 2022). This gap limits our understanding of how vestibular efficiency supports cognition under demanding conditions. To address this, the present study employed a novel “Multirule Visual Monitoring Task.” Adapted from vigilance paradigms (Funke et al., 2010; Nelson et al., 2014), this task simulates an air traffic control scenario where participants must simultaneously monitor for three distinct types of alerts based on multiple rules. By embedding concurrent decision rules, the task elevates cognitive complexity, providing an ideal context to examine how vestibular efficiency supports higher-order cognition when attentional resources are taxed (Lakatos et al., 2019; Allen et al., 2022).

In this study, vestibular function was quantified using the Modified Clinical Test of Sensory Integration and Balance (m-CTSIB), extracting a core metric reflecting vestibular compensation efficacy (Hupfeld et al., 2022). Key attentional functions were assessed using established measures, as attentional modulation is a core mechanism for integrating internal and external signals (Farb et al., 2012; Tune et al., 2018). Perceptual sensitivity was measured using the discrimination index (*d*′) from Signal Detection Theory. The central aim was to test the hypothesis that individual differences in vestibular function predict perceptual sensitivity under high cognitive load (Lacroix et al., 2021). Specifically, we examined whether vestibular function interacts with attentional breadth to enhance signal discrimination (*d*′), reflecting a more efficient allocation of neural resources.

It is crucial to note that the present design is correlational, examining associations rather than establishing causation. Consequently, mechanistic interpretations regarding the vestibular system as a cognitive anchor that directly optimizes resource allocation are presented as a plausible theoretical framework supported by prior literature, rather than as conclusions directly tested by the reported data.

The study was therefore designed to examine whether vestibular function is associated with perceptual sensitivity under high cognitive load and to assess its interaction with attentional breadth. The findings provide empirical evidence for a link between vestibular efficiency and performance in a complex cognitive task, thereby advancing the understanding of multisensory integration by clarifying the role of vestibular input in attentional resource allocation during complex perceptual decisions.

## 2 Materials and methods

### 2.1 Participants

A total of 98 science graduate students (M = 23.53 years, SD = 1.89 years) completed the Multirule Visual Monitoring Task. This group comprised 45 males (M = 23.84 years, SD = 2.03 years) and 53 females (M = 23.26 years, SD = 1.75 years). Independent samples *t*-tests and chi-square tests revealed no significant differences between male and female participants within the time–pressured condition in terms of age [*t* (96) = 1.52, *p* = 0.132] or group distribution [*χ^2^* (1) = 0.65, *p* = 0.419]. This study was approved by the Research Ethics Committee of Shaanxi Normal University (Approval No: 202516056). All participants provided written informed consent.

All participants were right-handed and had normal or corrected-to-normal vision. They reported no prior participation in experiments similar to the present study. Furthermore, clinical screening confirmed the absence of any history of dizziness, vertigo, or otological diseases, ensuring normal vestibular function.

### 2.2 Measure and procedure

Upon laboratory arrival, participants completed vestibular function testing; they then completed one randomly assigned attention assessment (attentional breadth or attention shift) in counterbalanced order, followed by both simple and difficult versions of the Multirule Visual Monitoring Task.

#### 2.2.1 Vestibular function assessment

Vestibular function was assessed using the Biodex Balance System (Biodex Medical Systems, Shirley, NY) via the Modified Clinical Test of Sensory Interaction in Balance (m-CTSIB). Participants maintained a standardized barefoot stance on the platform: left heel at coordinate F8, right heel at F14, medial malleoli 10 cm apart, toes angled 15° from the central axis, hands placed naturally on hips, and trunk upright. Static postural stability was quantified using the Stability Index (SI), where higher values indicate poorer stability. Four 30-s conditions were tested with 10-s inter-trial intervals: T1 (firm surface, eyes open), T2 (firm surface, eyes closed), T3 (foam surface with sponge pad, eyes open), and T4 (foam surface with sponge pad, eyes closed). The Vestibular Stability Index (VSI) was derived from SI values using Equation 1.


$$\mathrm{VSI}=({\mathrm{SI}}_{T2}-{\mathrm{SI}}_{T1})+({\mathrm{SI}}_{T4}-{\mathrm{SI}}_{T3})$$ (1)

#### 2.2.2 Attentional performance assessment

Attentional breadth and attentional shifting were assessed using a computerized task programmed in PsychoPy (v2023.2.3). Stimuli were presented on a 35.56 cm (14-inch) LCD monitor at 1,920 × 1,080 pixel resolution. Participants were seated 60 cm from the display screen during testing, with head position stabilized using a chin rest.

Attentional breadth was assessed using a visual enumeration task, which provides a quantitative estimation of the number of items one can apprehend in a single glance without serial counting. This method is grounded in the theoretical framework that links visuospatial attention capacity with working memory resources and the efficiency of early perceptual processing (Cowan et al., 2024). Neuroimaging evidence suggests that tasks requiring rapid quantity estimation engage a frontoparietal network associated with attentional control and visual short-term memory (Rosenberg et al., 2016). Therefore, while not exhaustive, performance on this task serves as a valid and widely-used operational index of momentary attentional capacity or the “spotlight” of attention (Hötting et al., 2021).

**Figure 1:**
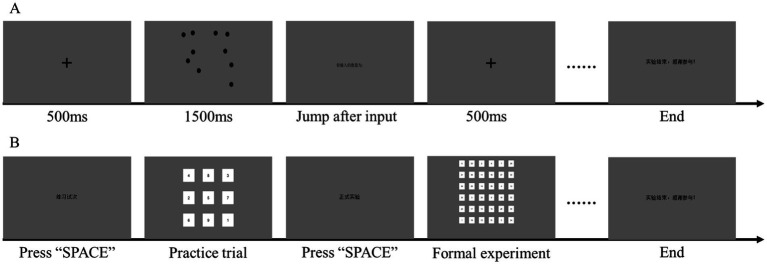
Attention measurement task protocols. **(A)** Attentional breadth task procedure; **(B)** attentional shifting task procedure.

The task comprised stimuli with 5–15 randomly distributed black circles. Each trial sequence initiated with a 500-ms black fixation cross (“+”), followed by target circle presentation; participants inputted perceived quantities via keyboard with responses computer-automated recorded, then pressed the spacebar to advance after 1,500-ms inter-trial intervals (Figure 1A). Following two practice trials, 20 formal trials were administered (one point per correct report, maximum = 20), with higher scores indicating greater attentional breadth capacity.

Attentional shifting, a core component of executive function involving the disengagement and re-orienting of cognitive focus between different tasks or mental sets, was measured using a computerized Schulte grid task. This task requires participants to continuously update their search target (the next sequential number) while suppressing interference from non-target numbers, thereby taxing cognitive flexibility, visual scanning, and inhibitory control—processes central to executive attention (Roberto et al., 2025).

The task (Figure 1B) consisted of a 6 × 6 matrix with randomly distributed Arabic numerals 1–36 (each number unique per cell). Participants clicked through the sequence 1-to-36 as rapidly as possible via mouse, with completion time automatically recorded. Two task sessions were administered, and attentional shifting performance was calculated as the mean reaction time across both sessions—shorter durations indicating superior shifting capacity.

#### 2.2.3 Multirule Visual Monitoring Task

**Figure 2:**
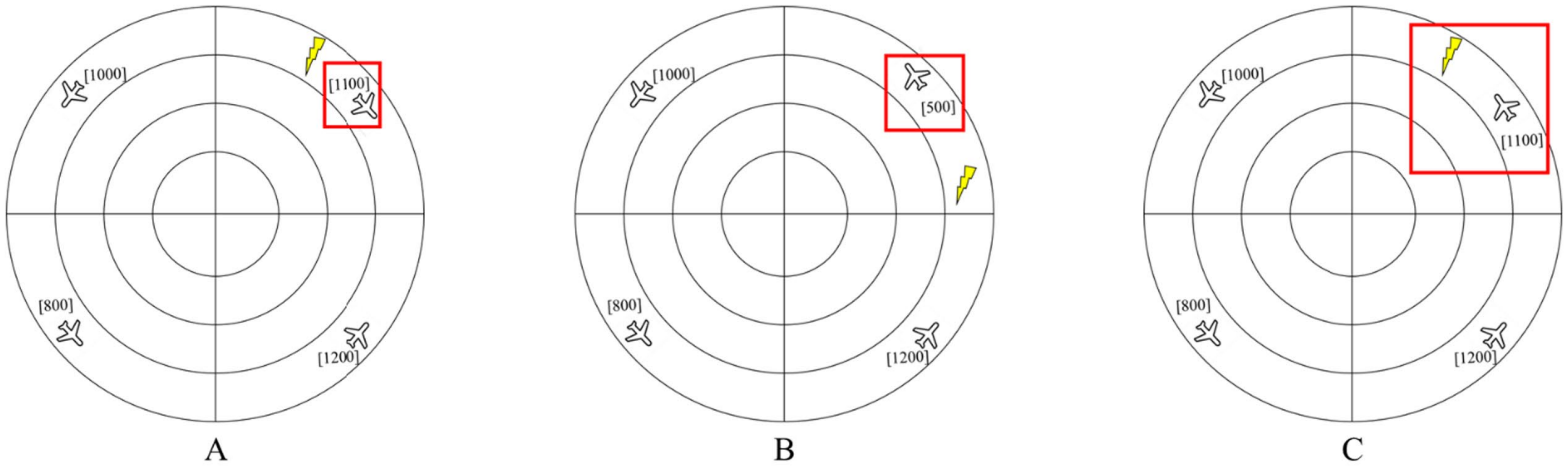
Schematic of the Multirule Visual Monitoring Task. **(A)** Short-term conflict alert; **(B)** minimum safe altitude warning; **(C)** restricted airspace warning.

The Multirule Visual Monitoring Task was employed to assess participants’ performance. Adapted from prior paradigms, the task comprised a simulated radar interface consisting of concentric circles (Funke et al., 2010; Nelson et al., 2014). Aircraft positions were represented by symbols, with adjacent numbers indicating their flight altitude (ft). Lightning symbols denoted restricted airspace zones. Three types of alerts were simulated: a Short-Term Conflict Alert, which was triggered when the separation between two aircraft fell below the minimum standard, indicating a potential conflict (Figure 2A); a Minimum Safe Altitude Warning, activated when an aircraft’s altitude descended below a predefined safe threshold (Figure 2B); and a Restricted Airspace Warning, issued upon an aircraft’s entry or projected entry into a restricted zone (Figure 2C). The reported Cronbach’s alpha coefficient of 0.931 demonstrates excellent internal consistency for the performance metrics, aligning with established psychometric reporting standards in cognitive task validation.

**Figure 3:**
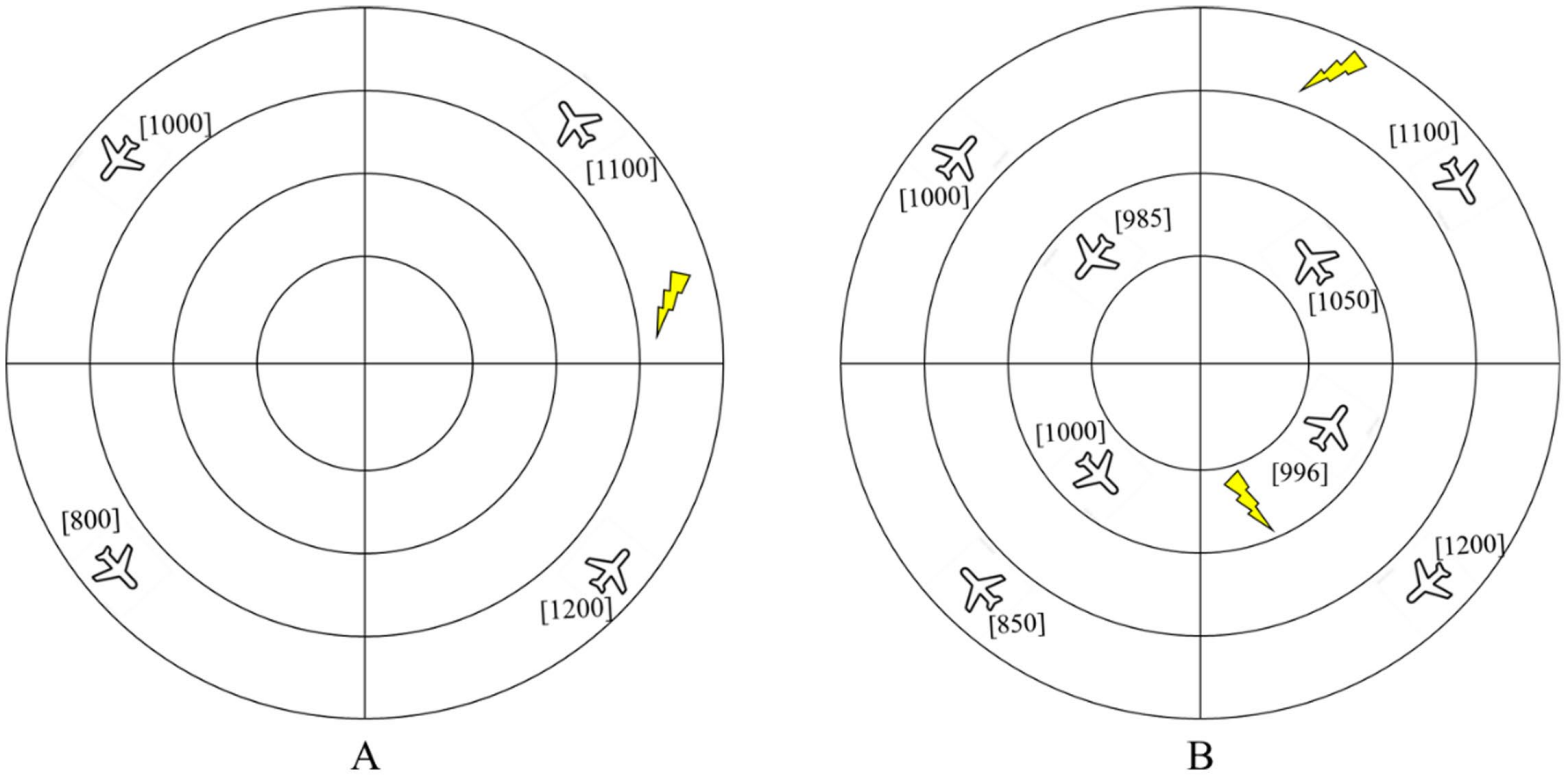
Radar plots of difficulty levels in the Multirule Visual Monitoring Task **(A)** simple mode; **(B)** difficult mode.

In the Multirule Visual Monitoring Task, participants were instructed to proactively detect potential threats based on three concurrent rules. For the Short-Term Conflict Alert, an alert was present if the aircraft headings were not uniformly clockwise or counter-clockwise. For the Minimum Safe Altitude Warning, an alert was triggered if any aircraft’s altitude was at or below 600 ft. For the Restricted Airspace Warning, an alert was generated if an aircraft occupied the same radial position as a lightning symbol (denoting restricted airspace) and was moving toward it. The activation of any alert indicated a hazardous state. The task included two difficulty modes: a simple mode with four aircraft (one per quadrant) and a difficult mode with eight aircraft (two per quadrant), as illustrated in Figure 3.

**Figure 4:**
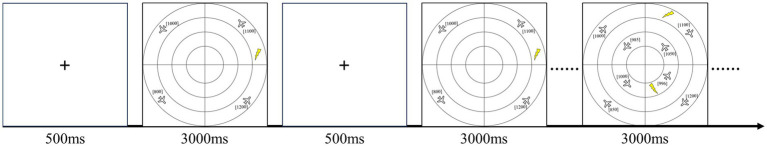
Workflow of the Multirule Visual Monitoring Task.

In the Multirule Visual Monitoring Task, dangerous scenarios (containing a single alert type) were presented at a 2:1 ratio against safe scenarios. Each difficulty mode (simple/difficult) included a 30-trial practice phase, which participants had to complete successfully before advancing to the formal test. The formal experiment comprised three 50-trial blocks with self-paced rests between them. A strict 3-s time constraint was set for each trial; a keypress within this period terminated the display immediately, while a lack of response led to an automatic advancement after 3 s (Figure 4).

Task reaction time and accuracy were recorded as performance metrics, and perceptual discriminability (*d*′) for safety/danger judgments was computed using signal detection theory (Equation 2).


$$d^{\prime }=Z(\mathrm{HR})-Z(\mathrm{FAR})$$ (2)

### 2.3 Design and statistical analysis

#### 2.3.1 Design

A between-subjects design was employed for the time–pressure Multirule Visual Monitoring Task. The design consisted of a 2 (vestibular function: stable, unstable) × 2 (attention dimension: [attentional breadth: high, low] or [attention shift: fast, slow]) × 2 (time pressure: present, absent) factorial structure. The dependent variable was the discriminability index (*d*′), calculated separately for the simple and difficult task conditions. The grouping criteria for the between-subjects factors are detailed in Table 1.

**Table 1:** Grouping criteria based on time variables.

Variable	Grouping criteria
Vestibular function	1.48
Attention shift	57.18
Attentional breadth	14.00

#### 2.3.2 Statistical analysis

Statistical analyses were performed using SPSS 26.0 (IBM Corp., Armonk, NY). The effects on Multirule Visual Monitoring Task performance were initially assessed with a multivariate analysis of variance (MANOVA). To examine the contributions of vestibular function and attention, task performance was dichotomized by median split and analyzed via binary logistic regression using the enter method. The discriminative ability of these factors was further evaluated by the area under the receiver operating characteristic (ROC) curve (AUC). Statistical significance was defined as a two-tailed *p*-value <0.05.

## 3 Results

### 3.1 The effects of vestibular function, attentional shifting, and attentional breadth on Multirule Visual Monitoring Task performance under time pressure

#### 3.1.1 Simple task condition

**Figure 5:**
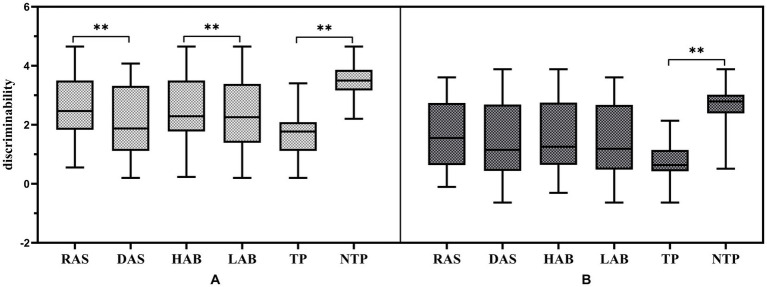
Effects of attentional shifting, breadth, and time pressure on discrimination capacity as a function of task complexity in the Multirule Visual Monitoring Task. **(A)**: Simple scenario; **(B)**: Complex scenario. RAS, rapid attentional shifting; DAS, delayed attentional shifting; HAB, high attentional breadth; LAB, low attentional breadth; TP, time-pressured; NTP, non-time-pressured. ^**^p < 0.01.

In the simple condition of the Multirule Visual Monitoring Task, significant main effects were observed for attentional shifting, *F*(1, 90) = 7.26, *p* = 0.008, *η_p_*^2^ = 0.075, and time pressure, *F*(1, 90) = 155.37, *p* < 0.001, *η_p_*^2^ = 0.633. No other main effects or interaction effects involving vestibular function were statistically significant (all *p* > 0.05; see Figure 5A).

A parallel analysis incorporating attentional breadth also revealed significant main effects for attentional breadth, *F*(1, 90) = 9.02, *p* = 0.003, *η_p_*^2^ = 0.091, and time pressure, *F*(1, 90) = 176.49, *p* < 0.001, *η_p_*^2^ = 0.662. Consistent with the previous analysis, neither the main effect of vestibular function nor any interaction effects involving vestibular function were statistically significant (all *p* > 0.05; see Figure 5A).

#### 3.1.2 Difficult task condition

Analysis of the difficult task condition revealed a significant main effect of time pressure, *F*(1, 90) = 281.55, *p* < 0.001, *η_p_*^2^ = 0.758, and a significant two-way interaction between attentional shifting and time pressure, *F*(1, 90) = 7.79, *p* = 0.006, *η_p_*^2^ = 0.080. Simple effects analysis indicated that attentional shifting significantly influenced performance under time pressure, *F*(1, 90) = 5.380, *p* = 0.023, *η_p_*^2^ = 0.056, but not in the no time pressure condition, *F*(1, 90) = 2.995, *p* = 0.087, *η_p_*^2^ = 0.032. No other main or interaction effects were significant (all *p* > 0.05; see Figure 5B).

When attentional breadth was examined in the difficult condition, a robust main effect of time pressure was again found, *F*(1, 90) = 272.045, *p* < 0.001, *η_p_*^2^ = 0.751. However, the main effects of attentional breadth and vestibular function, along with all associated interactions, were not statistically significant (all *p* > 0.05; see Figure 5B).

### 3.2 The independent and joint effects of vestibular function, attentional shifting, and attentional breadth on task performance

#### 3.2.1 Simple task condition

Performance on the simple Multirule Visual Monitoring Task was dichotomized into low (0) and high (1) discrimination groups based on a median split (*Mdn* = 2.26). A binary logistic regression was performed with this dichotomized performance as the dependent variable and vestibular function, attentional shifting, and attentional breadth as independent variables (Table 2). After adjustment for time pressure, a significant positive effect of attentional breadth (*p* = 0.038) and a significant negative effect of vestibular function (*p* = 0.032) on discrimination performance were observed. In contrast, the effect of attentional shifting was not significant (*p* > 0.05). These significant effects for attentional breadth and vestibular function persisted after additional adjustment for gender, while attentional shifting remained non-significant.

**Table 2:** Binary logistic regression predictors of discriminability in the simple Multirule Visual Monitoring Task.

Model	Variable	β	SE	Wald	0R (95%CI)	*p*
Model 1	Attention shift	−0.093	0.050	3.467	0.911 (0.827–1.005)	0.063
	Attentional breadth	0.239	0.115	4.307	1.270 (1.013–1.592)	0.038
	Vestibular function	−1.367	0.643	4.583	0.253 (0.072–0.890)	0.032
Model 2	Attention shift	−0.088	0.052	2.803	0.916 (0.826–1.015)	0.094
	Attentional breadth	0.248	0.122	4.136	1.282 (1.009–1.629)	0.042
	Vestibular function	−1.327	0.671	3.918	0.265 (0.071–0.987)	0.048

Model 1: adjusted for time pressure, with attention shifting, attentional breadth, and vestibular function entered separately; Model 2: adjusted for time pressure and gender, with attention shifting, attentional breadth, and vestibular function entered separately.

Subsequent binary logistic regression analyses were conducted to examine interaction effects, with the dichotomized performance as the dependent variable and the interaction terms (attentional shifting × vestibular function or attentional breadth × vestibular function) as independent variables (Table 3). Both interaction terms demonstrated significant effects after adjusting for time pressure (both *p* < 0.05). These significant interaction effects were maintained after further adjustment for gender (both *p* < 0.05).

**Table 3:** Binary logistic regression analysis of joint effects of vestibular function and attention on discriminability in the simple Multirule Visual Monitoring Task.

Model	Variable	β	SE	Wald	0R (95%CI)	*p*
Model 1	Attention shift	−0.114	0.055	4.307	0.892 (0.801–0.994)	0.038
	Vestibular function	−1.639	0.703	5.431	0.194 (0.049–0.771)	0.020
Model 2	Attentional breadth	0.321	0.135	5.630	1.379 (1.058–1.799)	0.018
	Vestibular function	−1.886	0.731	6.661	0.152 (0.036–0.635)	0.010
Model 3	Attention shift	−0.126	0.061	4.308	0.881 (0.782–0.993)	0.038
	Vestibular function	−1.765	0.755	5.469	0.171 (0.039–0.751)	0.019
Model 4	Attentional breadth	0.343	0.148	5.385	1.409 (1.055–1.882)	0.020
	Vestibular function	−1.873	0.763	6.019	0.154 (0.034–0.686)	0.014

Model 1: adjusted for time pressure, with attention shifting and vestibular function entered jointly; Model 2: adjusted for time pressure, with attentional breadth and vestibular function entered jointly; Model 3: adjusted for time pressure and gender, with attention shifting and vestibular function entered jointly; Model 4: adjusted for time pressure and gender, with attentional breadth and vestibular function entered jointly.

#### 3.2.2 Difficult task condition

Performance on the difficult Multirule Visual Monitoring Task was dichotomized using a median split (*Mdn* = 1.21). A binary logistic regression analysis, adjusting for time pressure, revealed that attentional breadth had a significant positive effect on discrimination performance (*p* = 0.038), while neither attentional shifting nor vestibular function showed significant main effects (*p* > 0.05; Table 4). After additional adjustment for gender, the significant positive effect of attentional breadth remained (*p* = 0.041), with attentional shifting and vestibular function continuing to show non-significant effects.

**Table 4:** Binary logistic regression analysis of discriminability predictors in the difficult Multirule Visual Monitoring Task.

Model	Variable	β	SE	Wald	0R (95%CI)	*p*
Model 1	Attention shift	−0.077	0.048	2.504	0.926 (0.842–1.018)	0.114
	Attentional breadth	0.239	0.115	4.307	1.270 (1.013–1.592)	0.038
	Vestibular function	0.481	0.600	0.643	1.618 (0.499–5.250)	0.423
Model 2	Attention shift	−0.072	0.050	2.075	0.931 (0.844–1.026)	0.150
	Attentional breadth	0.241	0.118	4.160	1.273 (1.009–1.605)	0.041
	Vestibular function	0.591	0.610	0.941	1.807 (0.547–5.968)	0.332

Model 1: adjusted for time pressure, with attention shifting, attentional breadth, and vestibular function entered separately; Model 2: adjusted for time pressure and gender, with attention shifting, attentional breadth, and vestibular function entered separately.

Further analyses modeling the interactions (attentional shifting × vestibular function and attentional breadth × vestibular function) were performed (Table 5). After time pressure adjustment, neither interaction term reached statistical significance (both *p* > 0.05). In the model containing the attentional breadth × vestibular function interaction, only the main effect of attentional breadth was significant (*p* = 0.043). These patterns persisted following gender adjustment: the interaction effects remained non-significant (both *p* > 0.05), and only the main effect of attentional breadth was significant (*p* = 0.050).

**Table 5:** Binary logistic regression analysis of joint effects of vestibular function and attention on discriminability in the difficult Multirule Visual Monitoring Task.

Model	Variable	β	SE	Wald	0R (95%CI)	*p*
Model 1	Attention shift	−0.076	0.049	2.447	0.927 (0.842–1.019)	0.118
	Vestibular function	0.479	0.620	0.595	1.614 (0.478–5.444)	0.440
Model 2	Attentional breadth	0.234	0.116	4.080	1.263 (1.007–1.585)	0.043
	Vestibular function	0.338	0.642	0.277	1.402 (0.398–4.939)	0.599
Model 3	Attention shift	−0.069	0.051	1.839	0.934 (0.846–1.031)	0.175
	Vestibular function	0.521	0.629	0.687	1.684 (0.491–5.772)	0.407
Model 4	Attentional breadth	0.232	0.119	3.831	1.261 (1.000–1.592)	0.050
	Vestibular function	0.430	0.647	0.441	1.537 (0.432–5.468)	0.506

Model 1: adjusted for time pressure, with attention shifting and vestibular function entered jointly; Model 2: adjusted for time pressure, with attentional breadth and vestibular function entered jointly; Model 3: adjusted for time pressure and gender, with attention shifting and vestibular function entered jointly; Model 4: adjusted for time pressure and gender, with attentional breadth and vestibular function entered jointly.

### 3.3 Discriminative ability of multisensory factors: vestibular function and attentional dimensions in task performances

Receiver operating characteristic (ROC) analysis was conducted to evaluate the independent and joint discriminative value of vestibular function, attentional shifting, and attentional breadth for performance on the simple Multirule Visual Monitoring Task. The predicted probabilities derived from the significant binary logistic regression models (which included vestibular function, attentional breadth, the vestibular × attentional breadth interaction, and the vestibular × attentional shifting interaction, adjusted for time pressure and gender) were used as test variables. The dichotomized discrimination performance (0 = low; 1 = high) served as the state variable. To statistically compare the discriminative ability of the different models, the areas under the ROC curves (AUC) were compared using the DeLong test for two correlated ROC curves.

**Figure 6:**
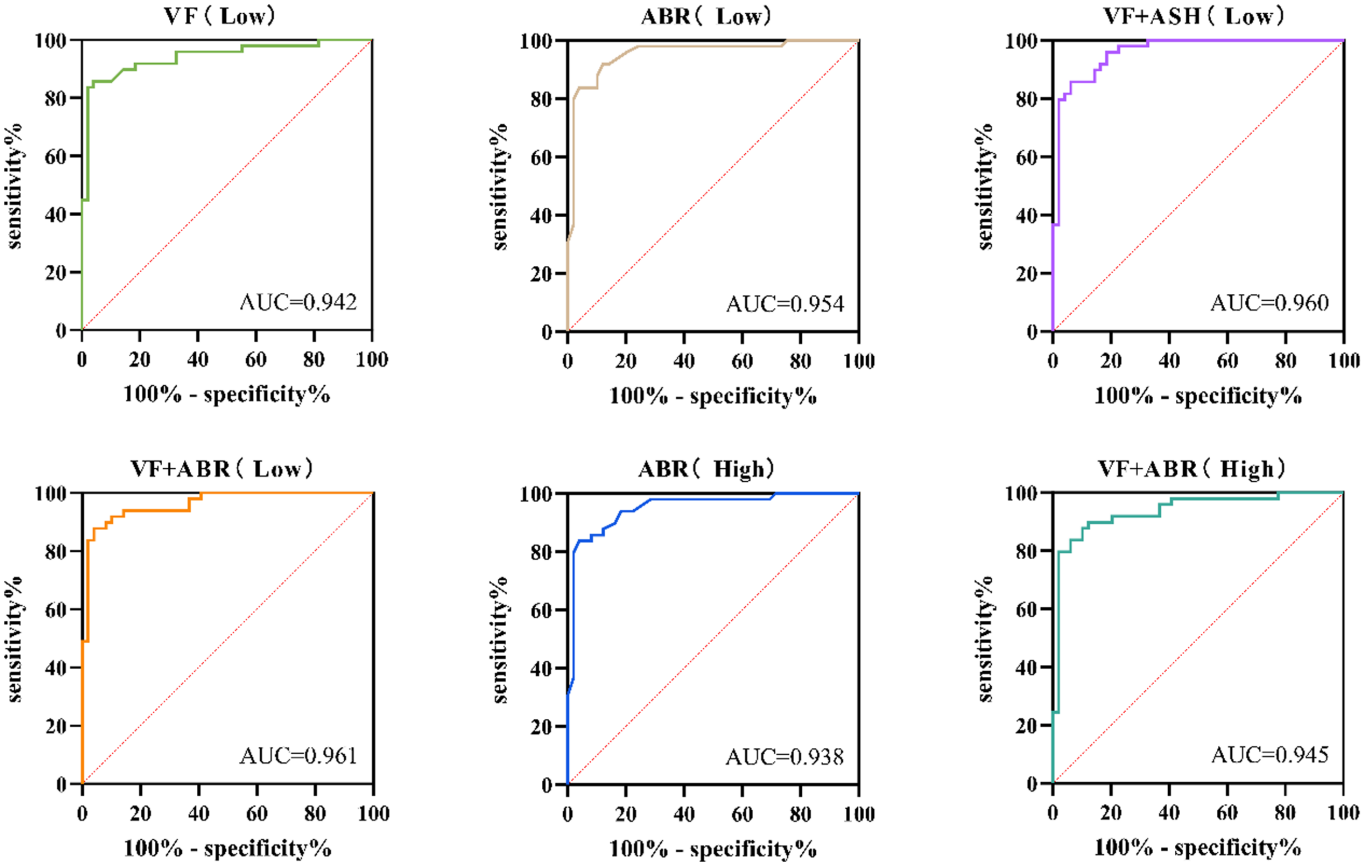
ROC curves for the individual and combined predictive value of vestibular function (VF), attentional breadth (ABR), and attention shifting (ASH) on discriminability in the Multirule Visual Monitoring Task. Low: simple scenario; High: complex scenario.

The analysis indicated that vestibular function, attentional breadth, the vestibular-shifting combination, and the vestibular-span combination all served as significant predictors of discrimination performance (all *p* < 0.001). Among these predictors, the model combining vestibular function and attentional breadth demonstrated higher discriminative performance (*AUC* = 0.961, *95% CI*: 0.927–0.996; *Youden’s index* = 0.837; *sensitivity* = 0.878; *specificity* = 0.959; Table 6; Figure. 6). However, no statistically significant differences were found between the areas under these ROC curves (all *p* > 0.05).

**Table 6:** ROC analysis and area under the curve comparison of vestibular function and attention assessment in the simple and difficult Multirule Visual Monitoring Task.

Task	Variable	AUC (95%CI)	Sensitivity	Specificity	*Z*	*p*
Simple	VF	0.942 (0.893–0.990)^*^	0.837	0.980		
	ABR	0.954 (0.911–0.996) ^*^	0.837	0.959	0.606	0.545
	VF + ASH	0.960 (0.925–0.995) ^*^	0.857	0.939	1.145	0.252
	VF + ABR	0.961 (0.927–0.996) ^*^	0.878	0.959	1.334	0.182
Difficult	ABR	0.938 (0.885–0.990) ^*^	0.857	0.980		
	VF + ABR	0.945 (0.883–0.988) ^*^	0.898	0.898	1.484	0.138

VF, vestibular function; ABR, attentional breadth; ASH, attention shifting; for the simple task, the *Z*-tests for comparing the areas under the curve were all conducted against VF, whereas for the difficult task, they were compared against ABR; ^*^p < 0.05.

For the difficult task condition, ROC analysis was performed using the predictors of attentional breadth and the vestibular-span combination (adjusted for gender and time pressure). Both predictors significantly forecast discrimination performance (both *p* < 0.001). Consistent with the simple task results, the model combining vestibular function and attentional breadth again showed higher discriminative performance (*AUC* = 0.945, *95% CI*: 0.927–0.996; *Youden’s index* = 0.816; *sensitivity* = 0.857; *specificity* = 0.959; Table 6; Figure 6). However, no statistically significant differences were found between the areas under these ROC curves (all *p* > 0.05).

## 4 Discussion

The present study examined the individual and combined contributions of vestibular function and attentional capacity to performance on a complex, multi-rule visual monitoring task. A key finding was that although an analysis of variance (ANOVA) performed on the continuous *d*′ revealed no significant Vestibular × Attention interaction, subsequent binary logistic regression and ROC curve analyses—which classified participants into high- and low-performance groups based on a median split—demonstrated that models combining vestibular function and attentional breadth were effective in classifying performance, with AUC values exceeding 0.93. This pattern of outcomes—specifically, a nonsignificant interaction in the ANOVA alongside effective classification in the logistic/ROC framework—suggests that the joint contribution of vestibular function and attentional breadth may be better suited to predicting categorical group membership than to explaining linear, incremental variations in performance scores. This observation offers a novel behavioral perspective that aligns with the conceptual view of the vestibular system as a “spatial cognitive anchor” (Pfeiffer et al., 2014), which may support efficient attentional deployment in complex environments.

The finding that vestibular function and attentional breadth jointly predicted high performance in the simple task condition is interpreted within a resource-optimization framework. It was hypothesized that efficient vestibular processing provides a stable, egocentric spatial reference, which could reduce the computational load associated with continuous spatial updating and context maintenance during the visual monitoring task. In such a scenario, conserved cognitive resources might then be allocated to support a broader, more flexible attentional strategy, as indexed by a larger attentional breadth. This interpretation aligns with dual-task studies demonstrating that postural or vestibular challenges consume attentional resources, thereby impairing concurrent cognitive performance (Bigelow and Agrawal, 2015; Chari et al., 2022). The present results extend this literature by suggesting that, conversely, more efficient vestibular function may help preserve attentional resources. This conservation potentially could potentially allows for the adoption of more demanding—yet more effective—attentional strategies under specific conditions.

Furthermore, these findings provide converging evidence from a healthy population for the broader “Cognitive-Vestibular Compensation Hypothesis.” While this hypothesis has traditionally been invoked to explain cognitive resource depletion in patients with vestibular dysfunction—who must compensate for unstable spatial signals—the current findings are consistent with its inverse proposition (Smith, 2017; Lacroix et al., 2021). Specifically, the results suggest that more efficient vestibular function may support performance under demanding conditions. This implies that the attentional and memory deficits observed clinically in vestibular patients may be partially attributable to the absence of a stable “spatial anchor.” Consequently, these patients are at an inherent resource disadvantage during tasks that require broad attentional engagement (Bigelow and Agrawal, 2015; Smith, 2022; Guo et al., 2024). Therefore, the present work could be viewed as extending the conceptual scope of this hypothesis from a model of “pathological compensation” to one of “performance support in health.” It highlights the role of the vestibular system not merely in restoring function after impairment but also in supporting cognitive performance under normative conditions. The joint association observed between vestibular function and attentional breadth may be underpinned by interactions within shared neural networks. Neuroimaging evidence indicates that the core vestibular cortical regions, such as the parieto-insular vestibular cortex (PIVC), are structurally and functionally connected with a fronto-parietal network critical for attentional control and spatial information processing (Frank and Greenlee, 2018; Dieterich and Brandt, 2024). It is plausible that a stable and precise vestibular signal could support the functional efficiency of this network, providing a neural substrate relevant for deploying attention over a wide spatial array. Furthermore, electroencephalography (EEG) studies have suggested that vestibular stimulation or postural sway can modulate oscillatory activity in alpha and theta bands, rhythms intimately linked to attentional alertness and cognitive control (Pepper and Nuttall, 2023). One speculative mechanism is that efficient input helps maintain a brain state conducive to the ‘wide-beam’ monitoring required by our task. While these neurophysiological findings offer a plausible framework for our behavioral results, direct evidence linking the specific vestibular and attentional measures used here to such neural mechanisms is lacking and represents a crucial avenue for future research.

The divergence between the ANOVA and logistic regression/ROC results warrants further methodological consideration. ANOVA tests for linear effects of predictors on the mean of a continuous outcome variable. In contrast, dichotomizing the performance variable and applying logistic regression shifts the analytical goal to evaluating the predictors’ efficacy in distinguishing between two distinct groups. This transformation can amplify the role of variables that act as enabling factors or threshold determinants rather than linear enhancers (Spencer et al., 2025). Our findings suggest that the vestibular-attention association may not manifest as a simple, dose–response improvement in d′ for all individuals. Instead, it may function as a combination associated with crossing a performance threshold into the higher-performing group. This distinction has practical relevance: identifying which individuals are likely to be high performers in complex, multi-tasking environments is a different, and often complementary, question to understanding how to incrementally improve everyone’s score. In this context, references to nonlinear or threshold-like patterns are intended to describe the structure of the observed associations, rather than to specify a formally tested mechanistic model.

The attenuation of the vestibular-attention association in the difficult task condition was an unanticipated finding. Nevertheless, a critical design limitation should be noted: the difficult task was always administered after the simple task, which fully confounded task difficulty with potential order, practice, or fatigue effects (Roberto et al., 2025; Sharpe and Tyndall, 2025). Consequently, the observed change in predictive patterns cannot be unequivocally attributed to increased cognitive load alone. It is plausible that accumulated mental fatigue altered the cognitive strategy or depleted the resources necessary for the potential performance benefits associated with efficient vestibular function. Alternatively, the increased rule complexity and time pressure in the difficult condition may have fundamentally altered task demands, potentially necessitating a more focused, resource-intensive attentional mode in which a broad attentional style becomes less advantageous (Staiano et al., 2024). Future studies must counterbalance task order or employ a within-subject design with difficulty as a formally manipulated factor to disentangle these possibilities.

Several important limitations of this study must be considered when interpreting the findings. First, the cross-sectional and correlational design precludes causal inferences regarding the observed relationships. Second, the sample consisted predominantly of young, healthy graduate students in STEM fields. This high-functioning, homogeneous cohort limits the generalizability of the findings to broader populations, including older adults or clinical populations with vestibular disorders, where such vestibular-cognitive interactions might be more pronounced or qualitatively different (Bigelow and Agrawal, 2015; Smith et al., 2024). Third, while the m-CTSIB and VSI provide ecologically valid assessments of vestibular contribution, they are not direct physiological measures of specific vestibular end organs. Future research would benefit from incorporating more precise clinical tools, such as the video head impulse test (vHIT) or vestibular evoked myogenic potentials (VEMPs), to obtain more objective and granular measures of vestibular integrity (Guo et al., 2024; Smith et al., 2024).

These limitations delineate clear directions for future research. To establish causality and explore underlying neural mechanisms, the following steps are warranted: (1) utilizing functional magnetic resonance imaging (fMRI) to directly investigate neural interactions between vestibular cortices and attention networks (e.g., the frontoparietal network) during task performance (Ibitoye et al., 2022); and (2) employing experimental manipulations such as galvanic vestibular stimulation (GVS) to transiently modulate vestibular function in healthy individuals, thereby testing its direct impact on performance in similar complex tasks and outlining a potential pathway for cognitive enhancement (Ferrè et al., 2012; Kaliuzhna et al., 2016; Blini et al., 2018).

Importantly, the promising assessment model derived in this study requires external validation. Its clinical utility should be tested directly within independent cohorts, particularly clinical populations diagnosed with vestibular disorders, to evaluate its potential as a screening tool for cognitive-vestibular profiles (Darwish et al., 2025). Finally, as noted previously, future experimental designs must rigorously control for order and fatigue effects, perhaps through fully counterbalanced designs or by including temporal covariates in statistical models, to isolate the pure effects of task difficulty.

## 5 Conclusion

This study provides evidence consistent with the view of the vestibular system as a contributor to cognitive processes. A combined effect of vestibular function and attentional breadth was observed, contributing to the evaluation of perceptual sensitivity under cognitive demand. These findings align with models of multisensory integration in which vestibular inputs may aid in refining neural resource allocation during complex tasks. Thus, the joint contribution of vestibular and attentional factors bridges domains of somatic stability and perceptual efficiency, advancing our understanding of cognitive adaptation. The vestibular system may therefore play a moderating role, supporting perceptual performance by helping to ground sensory processing in a stable spatial reference frame under demanding conditions. More broadly, the present study provides an empirical basis for generating testable hypotheses about how vestibular function and attentional breadth jointly contribute to individual differences in perceptual performance.

## Data Availability

The raw data supporting the conclusions of this article will be made available by the authors, without undue reservation.
